# Biofuel production from Macroalgae: present scenario and future scope

**DOI:** 10.1080/21655979.2021.1996019

**Published:** 2021-11-24

**Authors:** Godvin Sharmila V, Dinesh Kumar M, Arulazhagan Pugazhendi, Amit Kumar Bajhaiya, Poornachander Gugulothu, Rajesh Banu J

**Affiliations:** a Department of Civil Engineering, Rohini College of Engineering and Technology, Kanyakumari, India; b Department of Civil Engineering, Saveetha School of Engineering, Saveetha Institute of Medical and Technical Sciences(SIMATS), Chennai, India; c Center of Excellence in Environmental Studies, King Abdulaziz University, Jeddah, Saudi Arabia; d Department of Marine Biology, Faculty of Marine Sciences, King Abdulaziz University, Jeddah, Saudi Arabia; e Department of Microbiology, Central University of Tamil Nadu, Thiruvarur, India; f Department of Life Sciences, Central University of Tamil Nadu, Thiruvarur, India

**Keywords:** Biofuels, challenges, commercialization, cost, cultivation, Macroalgae

## Abstract

The current fossil fuel reserves are not sufficient to meet the increasing demand and very soon will become exhausted. Pollution, global warming, and inflated oil prices have led the quest for renewable energy sources. Macroalgae (green, brown, and red marine seaweed) is gaining popularity as a viable and promising renewable source for biofuels production. Numerous researches have been conducted to access the potential of macroalgae for generating diverse bioproducts such as biofuels. The existence of components such as carbohydrates and lipids, and the lack or deficiency of lignin, create macroalgae an enviable feedstock for biofuels generation. This review briefly covers the potential macroalgal species promoting the production of biofuels and their cultivation methods. It also illustrates the biofuel generation pathway and its efficiency along with the recent techniques to accelerate the product yield. In addition, the current analysis focuses on a cost-effective sustainable generation of biofuel along with commercialization and scaleup.

## 1. Introduction

The world’s largest fuel sources are rapidly and unpredictably diminishing as a result of significant result of rising population and growing needs with in renewable energy sector which is primarily associated with rapid urbanization and industrialization [1,2]. Fossil fuels are the primary, but not renewable source of energy. The indiscriminate utilization of fossil fuels leads to environmental impacts, poor air quality, and global climate change, which mostly contribute to ecological imbalance and health implications [3]. The demand for fossil fuels is anticipated to grow 40% from 2010 to 2040. Hence, providing a unique source of sustainable energy is a critical concern.

Among the promising alternatives of fossil fuels, various biomasses have shown significant progress and could be potential eco-friendly base products [4,5]. Biorefinery is the process of converting biomass into a variety of profitable products such as fuels and chemicals through conversion processes [6]. Biofuel is one such biorefinery energy product that is generated from biomass [7]. Based on the varieties of feedstock utilized, biofuels are categorized into first, second, and third generation fuels. First-generation biofuels are produced from feedstocks which are expended as food for human consumption. Wheat, sugarcane, rice and corn, sugar beet sorghum, and other crops fall into this category. It can be characterized as ‘conventional biofuels’ since it recovers renewable fuels using conventional technology. Second-generation biofuels are created in response to industrial and commercial applications such as expense, ineffectiveness, and also competitiveness with crop production. Mainly, the waste that is generated from this crop production is used for biofuels generation. Nonconsumable foods such as Agricultural residues and woody crops are utilized highly which are more difficult to extract and require sophisticated conversion technology.

Marine resources, seaweeds, and cyanobacteria are interesting sources for third-generation biofuel since it can produce better yields with less resource input. Macroalgae is perhaps the most potential non-consumable biofuel source as it can grow exponentially in saline water, adverse conditions, and in salty water. The algae biofuel is safe and extremely compostable and contains no sulfur [8]. All groups contain varying amounts of ash (18% – 55%), carbohydrates (25% – 60%), proteins (5% – 47%), and lipids (< 5%) which differ between species and are greatly influenced by biotic and abiotic habitat growth factors, such as temperature and light. Algae can even be transformed into a variety of fuels and it depends mostly on the technique and algal species used. Biofuels from algae are considered as third generation fuels and has advantages such as rapid growth, high CO_2_ capture, and ease of cultivation even in barren lands which has the potential to meet energy crisis [9]. The algal biomass contains substances such as acyl glycerides and fatty acids which are used in biofuel production, thus lessen fossil fuels usages. The oil extracted from algae can be used for biodiesel production and the residual biomass obtained are rich in sugar content that can be used for bioethanol production [10,11].

However, several challenges need to be tackled to allow commercial biofuel production from algae in scaleup and are sufficient to make a significant contribution to energy requirements. The objective of this review is to discuss several biofuel productions approaches from macroalgae. Also, the Patent of macroalgal biofuel generation has been discussed to achieve in-depth knowledge. This article concludes with a discussion of certain hurdles which prevail in macroalgal biorefineries, along with forthcoming research areas which should be examined for future industrial expansion.

## 2. Macroalgae – feedstock for biofuel production, cultivation methods, and its environmental impact

Macroalgae is a diverse and non-phylogenetic macroscopic aquatic eukaryote that belongs to *Rhodophyta* (red algae), *phaeophyta* (green algae), and *Phaeophyceae* (brown algae) [2]. Algae can be cultivated in almost all types of water including wastewater [12]. Moreover, the algal growth rate is about 20–30 times quicker than fodder crops and the oil content present in macroalgae is around 30 times more than the conventional feedstocks [13]. The algal source is completely biodegradable and sulfur free, the oil derived from algae has better quality [14]. Further, the absence of lignin makes the macroalgae easy to digest by microbes in the biorefinery process [15] and makes it easier to convert into a biofuel than land-based plants [16]. Biomass residues after the conversion processes can be used for heating purposes, fertilizers, and other types of fuel production [17]. Macroalgae have water content with rich carbohydrates (25% – 50%), protein (7% – 15%), and lipid (1% – 5%) [18] which makes macroalgae a promising feedstock for biodiesel production, bioethanol and biohydrogen production [19]. Similarly, macroalgae can also be used as food supplements [20], hydrocolloids, healing materials, fertilizer, and animal feed. In the food industry, macroalgae account for $5 billion worldwide on an annual basis, which is 83% – 90% of the total seaweed industry. Many researchers have studied about the usage of macroalgae as a feedstock for biofuel production such as biodiesel [21,22], bioethanol [23–25], biohydrogen [26–28], biomethane [29–31] and bio-oil [32,33]. Most of the results showed a positive review about the production of biofuels from macroalgae.

The macroalgae biomass market is expanding both in market capitalization on an annualized basis, with statistical information from the Food and Agriculture Organization of the United Nations (FAO) indicating that global macroalgal biomass production in 2016 amounted to approximately 30 million tonnes at a value of USD$ 11.6 billion. Asia is the world’s greatest producer of macroalgae, with China leading the way with 14 million tonnes valued at USD$ 8.6 billion, followed by Africa with roughly 140,000 tonnes and the Americas with 15,634 tonnes. A variety of macroalgal species, including *Laminaria japonica, Eucheuma spp., Kappaphycusalverezii, Pyropiayezoensis, Undaria pinnatifida, and Graciliariaverrucosea*, have already been mass cultured in Asia [34]. Europe, on the other hand, still has a limited aquaculture industry, and cultivation methods are lagging. Nonetheless, the effort to stimulate the European macroalgae market and aquaculture industry is in its early stages, and both academic and commercial interests have propelled strategies to farm macroalgae on a bigger scale.

**Figure 1.**
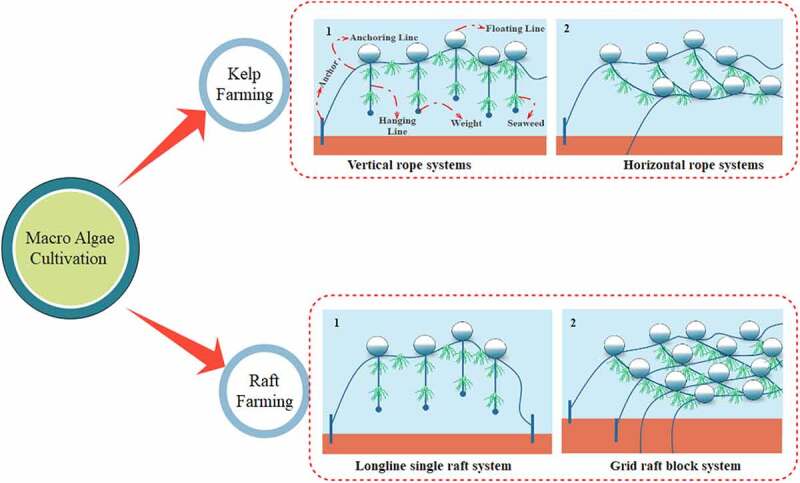
Various methods of Macroalgae cultivation system

The macroalgal can be cultivated both in offshore and onshore in various methods. The offshore cultivation includes kelp growth, raft cultivation, and floating cultivation [30] which is shown in Figure 1. Due to less consumption of cost for installation and maintenance, cultivation using ropes or nets is considered to be a prevalent cultivation technique. Lagoons are used for culturing macroalgae in which the nutrients are available from seawater. Fixed off bottom, long lines and rock-based farming are the other methods used in macroalgae cultivation. Transplantation is another cultivation method in which species saplings are allowed to be grown indoor, later they are cultured in the tanks and finally transplanted into the sea using ropes [35].

In Onshore cultivation techniques, seawater has been extensively used for cultivation and has the advantage of prohibitive extend of control over safety and high product yield. It offers high adaptability for a wider range of macroalgae and is more sustainable than offshore cultivation since marine species are not affected by onshore cultivation. In addition, mixing is a potential factor that promotes better algal growth in this type of cultivation. Proper agitation or circulation have been preferred to mix algal cultures effectively but consume more cost. So far, this onshore cultivation lacks a sustainable low-cost innovative approach for implementation on large scale. It is also possible that a biotic and abiotic ecosystem could get intruded upon by open farming. This reduces the grade of the algae, making it unsuitable for use in the pharmaceutical, chemical, and cosmetic industries [36]. A ring-shaped culture technique for algal cultivation on land was developed by Sebok *et al*. [37]. Through this strategy, expenses were drastically decreased by lowering the level of cultivation medium required [38]. In addition to supplying CO_2_ and nutrients individually, this method also absorbs the heat during agitation, leading to a more efficient growth phase. Moreover, the growth rate of the cultivated algal biomass is important that shows the impact of the cultivation methods. Yong *et al*. [39] determined the standard formula for calculating the growth rate of the algae, then the formula as follows:

(1) $$Growth\;rate\;\left(G\right)=\left[\left(Wf/Wi\right)\;1/T-1\right]\;x\;100\%$$

Where,

Wi – Initial weight of algal biomass,

Wf – Final weight of algal biomass and

T – Number of days in culture

The growth rate of the algal biomass was helpful in the assessment of better and essential nutrient-rich algae which promotes better biofuels generation. To cope with this high rate of growth, the biofuel industry and governments are constantly exploring new biofuel feedstocks, processing technologies, and policy mechanisms in order to ensure that future expansion is achievable and sustainable. Grown algae are measured by weighing the drained algae *thalli* at the beginning and at the end of the test.

Macroalgae offer a good unique atmosphere for marine organisms to sustains and fosters ecosystems [40]. Light intensity, turbidity, water temperature, nutrient concentrations, pH, and salinity are all factors that affect algae growth. But, algal harvesting causes damage to the ecosystem and becomes an issue [41]. In addition, Inorganic fertilizers like nitrogen and phosphorus has been used to flourish the growth of macroalgae. This nutrient enrichment induces algal blooming which could be seen in coastline which disrupts the ecosystems of its surroundings and probably results in hypoxia [42]. However, in the deep sea, this consequence is decreased. In addition, a substantial percentage of inorganic carbon is captured by macroalgae during photosynthesis and will be first metabolized as carbon dioxide and then as HCO^−3^and again to carbon dioxide. Using the process such as carbon trapping, photorespiration, and respiration, carbon will indeed be returned to saltwater. Through biological degradation, a component of the carbon is converted to carbon dioxide, while the residue persists as particulate organic carbon in the ocean, where it eventually settles on the bottom. Macroalgae have the last opportunity to capture phosphate until it gets diluted in deep waters. Seaweed farming has been progressively used as a promising nutrient removal technology [43]. Furthermore, a reduction in irradiance throughout the aquatic environment beneath macroalgae culture sites may have a deleterious influence on other marine creatures in shallow areas. Macroalgae in integrated multitrophic aquaculture can employ nutrients for fish farming as fertilizers in algae grown both in land-based and offshore marine culture systems

## 3. Biofuel production from Macroalgae

**Figure 2.**
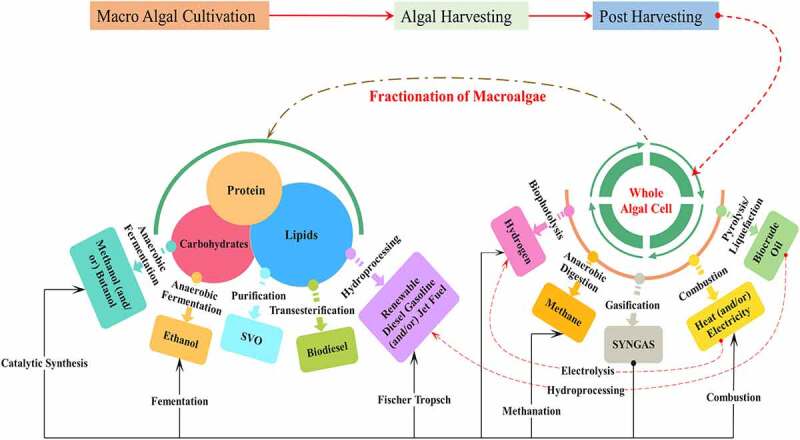
Macroalgal biofuel refinery

Biofuels are any solid, liquid, or gaseous fuels that are obtained from biological matter. These biofuels are capable of being used in automobiles and a variety of industrial activities. First, second, and third-generation biofuels are dependent upon the type of biomass. Biofuels can be derived from macroalgae through various biochemical and thermochemical methods which are shown in Figure 2. The most commonly used processes for the production of biofuels are transesterification, liquefaction, fermentation, anaerobic digestion, and pyrolysis. However, the complex structure of the algal biomass may affect the hydrolysis process which is a rate-limiting step that consumes more time. This affects the biofuel yields; hence it can be reduced by introducing suitable pretreatment [44]. Pretreatments break the bond of molecules and depolymerize the complex structure, thus increasing the solubilization [45]. The solubilized samples can be easily used in the conversion process and also enhance biofuel production. Various pretreatment methods such as physical, chemical, biological, mechanical, and combinative methods are used for solubilization of the complex substrate in macroalgae. Biofuel production from various macroalgal species is listed in Table 1.

**Table 1.** Biofuel production from macroalgae

Type of Biofuel	Species Type	Pretreatment methods orConversion techniques	Pretreatment or conversion technique conditions	Biofuel Yield or production potential	References
Biodiesel	*Ulva fasciata*	Catalytic transesterification	Molar ratio of methanol: oil – 9:1Time – 6 hoursTemperature – 80-100°C	88%	[17]
	*Chaetomorpha antennina*	Transesterification	Chloroform-ethanol solvent- 1:20 (w/v)	2.1 mL/10g_biomass_	[46]
	*Gracilaria corticata*	Transesterification	Hexane-ether solvent – 1:20 (w/v)	2 mL/10g_biomass_	[46]
	*Ulva intestinalis*	Transesterification	-	32.3 mg/g dw	[47]
	*Enteromorpha compressa*	Base transesterification	Base – 1% NaOH,Methanol–oil ratio – 9:1, Temperature – 60°CTime – 70 min	90.6%	[58]
Bioethanol	*Chaetomorpha linum*	Wet oxidation method	Temperature – 200°C	44 g ethanol/100 g glucan	[75]
	*Saccharina japonica*	Low acid pretreatment	Acid – 0.06% (w/w) sulfuric acidTemperature – 170°CTime – 15 min	6.65 g/L	[22]
	*Saccharinajaponica*	Thermal acid hydrolysis	Acid – 40 mM H_2_SO_4_Temperature – 121°CTime – 60 min	7.7 g/L	[72]
	*Laminaria digitata*	Oven drying	Temperature – 70°C,Time −72 h	13.6 ± 0.2 μL/gDS	[79]
	*Ulva linza*	Mild acid hydrolysis	Acid condition – 3% H2SO4	12.01%	[102]
Biohydrogen	*Laminaria japonica*	Microwave	Temperature – 160°C,Time – 30 min	15.8 mL/g TS	[59]
	*Laminaria japonica*	Ultrasonic	Sonication frequency – 20 kHz	23.56 ± 4.5 mL/g	[60]
	*Laminaria digitate*	Hydrothermal	Temperature – 140°CTime – 20 min	44.0 ± 1.2 mL/g VS	[61]
	*Chaetomorpha antennina*	Surfactant-aided microwave pretreatment	Microwave power – 0.36 kWAmmonium dodecyl sulfate – 0.0035	74.5 mL/g COD	[25]
	*Ulva reticulate*	Microwave-H_2_O_2_ alkali pretreatment	Microwave power – 0.36 kWH_2_O_2_ dosage – 24 mg H_2_O_2_/g biomasspH – 10	87.5 mL H_2_/g COD	[77]
Biomethane	*Palmaria palmata*	Anaerobic digestion	Semi continuous anaerobic digestion	320 mL CH_4_/gVS	[2]
	*Chaetomorpha antennina*	Ozone disperser pretreatment	Disperser g force – 1,613 g,Treatment time – 30 min,Ozone dosage – 0.00049 g O_3_/g TS	0.20 g COD/g COD	[30]
	*Chaetomorpha antennina*	Thermo-chemo disperser	Disperser g-force of 1613 g,Temperature – 80°C,NaOH – 1 N,pH – 11	215 mL/g VS	[64]
	*Laminaria digitata*	Heat	Temperature – 104°C	Dried biomass – 97.66 m^3^ CH_4_/tFresh biomass – 67.24 m^3^ CH_4_/t	[76]
	*Laminaria digitata*	Oven drying	Temperature – 70°C,Time −72 h	235.4 ± 14.1 mL/gVS	[79]
Bio oil	*Saccharina japonica*	Fixed bed reactor pyrolysis	Temperature – 450°C	47% conversion	[70]
	*Ulva lactuca*	Microwave pyrolysis	Temperature – 500°C	18.4 wt.%	[81]
	*Porphyra tenera*	Packed tube reactor pyrolysis	Temperature – 500°C	47.4 wt.%	[69]
	*Laminaria japonica*	Packed tube reactor pyrolysis	Temperature – 500°C	45.8 wt.%	[69]
	*Undaria pinnatifida*	Packed tube reactor pyrolysis	Temperature – 500°C	37.5 wt.%	[69]

### 3.1. Biodiesel generation

A mixture of monoalkyl esters of long-chain fatty acids extracted from algal biomass is biodiesel. Comparing biodiesel to fossil fuels, it has exceptional ignition properties and lowers fumes and carbon dioxide emission levels by 78% [46]. Osman *et al*. [47] studied biodiesel production from *Ulva intestinalis* and recovered a yield of 32.3 mg/g dw. Sharmila *et al*. [48] studied biodiesel production from *Chaetomorphaantennina* and *Gracilariacorticata*, achieving a biodiesel content of 2.4 mL and 2 mL per 10 grams of algal biomass. Tamilarasan [49] esterified the FFAs of *Enteromorpha compressa* algal oil from 6.3% to 0.34%, and then two steps are developed for biodiesel production. During the first step, the FFAs were established with an acid catalyst, and then the oil is turned into biodiesel in the second step. Another attempt used *Cladophora glomerata* to produce glucose and then converted glucose to free fatty acids for biodiesel. Recently, Xu [50] attempted using macroalgae as a carbon source of oleaginous yeast to produce biodiesel, and the maximum lipid content was 48.30% meanwhile the by-product FFAs accompany mannitol can be used to culture the oleaginous yeast.

### 3.2. Bioethanol production

Bioethanol production from *Sargassum spp*. was carried out by Borines *et al*. [51] with a conversion yield rate of 89%. Using fermentation, *Gracilariaverrucosa*, red seaweed is used to produce bioethanol production with a yield of 0.43 g/g sugars was achieved [52]. Yoza and Masutani [53], experimented with the bioethanol production from macroalgae biomass, *Ulva reticulate* in which 0.37% v/v concentration of bioethanol is produced from 1 gram of sample. The authors also reported the above results to correspond to approximate 90 liters of ethanol yielded per dry tonne of macroalgae. A study by Osman *et al*. [47] on bioethanol production from *Ulva intestinalis* recovered a yield of 0.081 g/g dw. Bioethanol conversion yield of 90.9% was obtained through saccharification and fermentation methods by treating seaweed waste [54]. In a batch reactor, anaerobic fermentation using B. *Custersii* generated 11.8 g/L ethanol from 90 g/L sugar whereas about 27.6 g/L ethanol from 72.2 g/L sugar in a continuous reactor [55]. Also, results from Offei *et al* [22] concluded that E. *Cottonii* could be a potential feedstock for bioethanol production. Red algae, *Palmariapalmata*, mainly containing carrageenan, released glucose, galactose, and sugars by acid hydrolysis (0.4 M H_2_SO_4_ at 125°C for 25 min) and then were fermented to ethanol [56]. *Kappaphycusalvarezii* [57] biomass was saccharified at 100°C in 0.9 M H_2_SO_4_ and the best yields for saccharification were 26.2% and 30.6% (w/w) at the laboratory (250 g) and bench (16 kg) scales, respectively. Stefan Kraan et al. [58] reported that washing macroalgae in acidic water (0.09 M HCl in H_2_O) at 65°C enhanced hydrolysis of *laminarin*.

### 3.3. Biohydrogen production

Biohydrogen is considered as a clean sustainable energy with a high-energy yield and it is the main source of future fuel. Hydrogen yield of 109.6 mL/g COD (Chemical Oxygen Demand) was achieved by treating *Laminaria japonica* using heat treatment at 170°C [59]. Yin *et al*. [60] experimented with microwave pretreatment for treating macroalgae *Laminaria japonica* at temperature 160°C for 30 min and obtained a hydrogen yield of 15.8 mL/g TS. Using disperser treatment, Kumar *et al*. [24] achieved biohydrogen production of 45.5 mL by treating algal biomass, *Ulva reticulate*. Algae *Laminaria japonica* is treated using an alkaline treatment which yielded 15 mL/g of biohydrogen [61]. Also, biohydrogen yield of 63 dm^3^/kg VS was obtained by treating the macroalgae using hydrogen peroxide chemical [62]. Yin and Wang [63], studied the combined microwave and acid pretreatment method to *Laminaria japonica* and achieved biohydrogen production of 28 mL/g TS at 140°C with 1% H_2_SO_4_ in 15 min.

### 3.4. Biomethane production

Biomethane production of 47.25 mL/g COD was obtained by treating the algal biomass, *Chaetomorphaantennina*, through chemo disperser treatment [64]. Jard *et al*. [65] studied biomethane production by treating *Palmaria palmate* a red macroalgae, and achieved high biomethane production of 308 ± 9 mL/gVS. Gurung *et al*. [66] studied the biomethane production from green and brown algae and obtained 256 ± 28 and 179 ± 35 mL/g VS biomethane as yield respectively. Biomethane yield of 70% was achieved by treating *Laminaria hyperborean* using anaerobic digestion [67]. Marine biomass has shown promise for stable methane production, yielding between 140 mL and 280 mL of methane per g volatile solids (VS) for green and brown algae genera, such as *Sargassum, Gracilaria, Laminaria, Ascophyllum*, and *Ulva*. Some studies even suggest biomethane recovery of 260–500 mL methane per g VS for *Laminaria sp., Macrocystis sp*., and *Gracilaria sp.*

### 3.5. Bio-Oil production

Bio-oil can be directly used for fuel internal combustion engines and also used as a chemical. Pyrolysis is considered to be one of the most possible conversion processes to produce bio-oil by heating algal biomass in absence of oxygen. The Hydrothermal liquefaction of the green macroalgal species *Enteromorpha prolifera* yielded of bio-oil of 23.0% dw (energy density of 29.89 MJ/kg) at 300°C, 30 min in the presence of Na_2_CO_3_ as catalyst. Similarly, Anastasakis and Ross [68] investigated the same liquefaction in brown macroalgae *Laminaria saccharina* which infiuences reaction parameters and yielded the highest bio-crude of 19.3% having algal/water ratio as 1:10 at 350°C and a residence time of 15 min without catalyst. Dong *et al*. [69] investigated bio-oil production from macroalgae using a fixed-bed reactor and yielded 47% with 33% of biochar as its co-product. Wang *et al*. [70] reported bio-oil production from macroalgae using microwave treatment and achieved a maximum yield of 18.4 wt.%.

## 4. Recent approaches in enhancing biofuel generation

Nanotechnology is an emerging technique to enhance biofuel production in various sectors which is gaining importance [71,72]. Its role in macroalgal biorefinery is to synthesis nanoparticles for shifting components within the algal biomass due to high surface area. A recent approach in enhancing the biofuel generation from macroalgae is the immobilization of cellulase by nanoparticles which could minimize the hydrolyze enzyme consumption [73]. Also, the bio-synthesis of magnetic nanoparticles in the field of biofuel production is in progress. Macroalgae which is a bio-nano factories have a remarkable capacity in producing metallic nanoparticles in both wet and dry forms. To produce cost-effective and sustainable biofuels, integrated approaches are formulated. Earlier research has shown that the conductive nanomaterial graphene can improve AD performance by stimulating direct interspecies electron transfer in intricate marine communities. On the contrary, excess graphene is attributed to the microbial inhibition caused by a high concentration of nanoscale. Despite the considerable enhancement in biomethane production, the high cost of graphene can be an obstacle to its practical application. Mainly, the processes such as pyrolysis, fermentation and hydrothermal liquefaction, etc., are preferred for integrated techniques. Integration anaerobic fermentation and pyrolysis enhance the methane yield by 17% and bio-oil yield by 10% [74]. Another promising technology in the field of biofuel production is hydrothermal liquefaction. Nearly 80 to 85% of moisture content in the macroalgal species is rapidly exhausted for fuel generation during hydrothermal conversion or liquefaction. Also, this hydrothermal liquefaction is combined with microwave for generating cost-effective fuels through depolymerization of the algae into sugars. An ideal and optimal environment can be created by microwave pyrolysis to produce biofuels from macroalgae for the aviation sector [75]. Integration anaerobic fermentation and pyrolysis enhance the methane yield by 17% and bio-oil yield by 10%. Also, agar extraction before anaerobic digestion of algal biomass enhances biomethane and biodiesel generation. Genetic and metabolic engineering approaches have recently gained importance in macroalgal biorefinery, offering an upsurge in biotechnology and cutting-edge tools [76,77].

## 5. Cost and economics

The economics of biofuel production from macroalgae is critical in creating sustainable cultivation, harvesting, and usage [78,79]. Due to the consumption of expenses for high labor, expensive equipment, and supplies in various algal farming, the profit obtained in the form of biofuel as an outcome is ought to be significant enough to make it viable. Even though the biofuel generation from macroalgae is extensively focused by the researchers, it is limited compared to the potentially serious impact in predicting yield and pricing the derived products (biofuels). The factors such as pretreatment and processing of macroalgae biomass, as well as seasonal variations may change its composition and have a significant impact on the biofuel yield. Owing to the high potential of algal product production in the field of food and medicinal products, there needs extensive research in its characteristics. But only meager studies are targeted on its economic assessment [80,81] due to the uncertainty in profit of algal biorefinery which was listed as follows:

- Organic feedstocks – Algal Species selection, Product obtained in varied climate and sea conditions
- Basic Requirements – Labor, Equipment, and supplies in various algal farming
- Demand – New product introduction, market conditions, and price of substitutes
- Investment – High capital cost and lack of public policies
- Processing Technology – Immature, complex, scalability, and cost
- Contracting – Assymteric info, logistics and transportation, and quality.

However, there are several cost-effective methods of cultivating macroalgae, such as generating useful derivatives such as biogas, biohydrogen, bio-oil, biodiesel etc., or combining macroalgae farms with many other aquaculture farms. To create a profitable macroalgae farm, it is necessary to offer a good price for the products derived on wet basis as €2/kg algae. Otherwise, integrated hatchery development to produce a relatively valued invertebrate, such as scallops along with algal cultivation. Concerning the significance of derived biofuels, macroalgae culture is about to gain attraction on an international market. One of the major rate-limiting factors is the high expense of cultivating macroalgae. Currently, the probable cost of producing fuel from macroalgae is considerable. Though manufacturing costs are high, when the sector expands, expenditures might quickly drop due to increased efficiencies and scalability. Besides that, the expenditure of converting into biofuels or bioenergy indeed should be taken into consideration in economic assessment. Three technologies were compared *viz*., methane generation with a possible contribution in fuel through the use of syngas and methanol; macroalgae fermentation into ethanol; and hydrothermal liquefaction into liquid fuels. During the biofuel generation from *Ulva* species, the cost of $ 2.21/ kg could be consumed where the revenue of the *ulvan* component was ranging from $8–10.4/kg [82]. The detailed techno-economic assessment of macroalgae biorefining reflects the cost of equipment and operations such as overhead expenses, cost index of Chemical Engineering plant, supply of feedstock and reagents usage, along with scaling factors. This revealed the production cost as US$3.7 million annually, having *Ulvan* prices as US$395/g, whereas the overall production expense was valued to be US$1.2 million [82]. As a whole, handling expenses (US$2.01/ kg to US$2.21/kg) are consistent. This similarity in total handling expenses (US$2.01/kg to US$2.21/kg) obscures the primary difference in pricing the *Ulvan*. However, Prabhu *et al*. [83] linked this to marketable carrageenan costs while Sadhukhan *et al*. [84] appear to have analytical grade material as reference. This variation in value is emphasized through the nonexistence of commercialization of this polysaccharide in comparison to alginate or carrageenan, highlighting the difficulties in estimating techno-economic analysis for innovative processing methods. For each system, the permissible limits of feedstock expense for algae were calculated to be $6- $28 per gallon.

Furthermore, the processing of algae may generate significant food products such as alginate and agar [85]. Protein extraction from macroalgae biomass is widely studied; however, the additional market is dominated by algal goods, which have proved to be an economically viable alternative to other marine protein resources [86]. Chemicals such as levulinic acid, 2,5-furandi-carboxylic acid, succinic acid, and lactic acid have been proposed as possible biorefinery products, however, the feasibility of the process varies depending on the chemicals generated. The costs of the mixtures remain heavily influenced by both the purity and the market requirement for these goods.

## 6. Commercialization and scale-up

Since several biochemical components of macroalgae have established commercial utility already, the advent of the aforementioned potential of macroalgal biorefineries has clearly emphasized the possibility of producing novel bioproducts from macroalgae [87]. Even though these bioprocesses are only in progress, the yield of such biofuel goods gives substantial commercialization as well as product allocation opportunities.

Macroalgae is chosen to be a primary feasible feedstock for the production of biofuels and extra biochemicals *via* biorefinery operations. The production of biofuels from macroalgal biomass is still in the developing stage due to its economical consideration of the techniques utilized for processing and the intermediates obtained during the biorefinery process has gained popularity. The major types of biofuels usually produced from macroalgae include biomethane, biobutanol, bioethanol, and biodiesel. Further in addition to this bio-fuels including certain hydrocarbon derivatives such as bio-oil have become fewer prevalent, even if studies persist. The rapid outgrowing of liquid biofuels namely biobutanol, bioethanol, and biodiesel is an effort to engage these biofuels in the transport sector to substitute petrol and diesel to increase government incentives. Bioethanol, particularly, has been regarded as perhaps the most significant biofuels. However, a great deal of solutions has been proposed on the development of macroalgal bioethanol, since there found a complexity while using conventional fermentation along with bacterial strain to convert macroalgal polysaccharides and monosaccharides to bioethanol which is a major impediment to its commercialization. Research has been carried out to produce strong stresses using metabolic and bioengineering techniques using the intrinsic macroalgal monosaccharides for bioethanol production. Conversely, a few hypothetical procedures of biorefining create a residue enriched cellulosic material, which is then hydrolyzed and then fermented to bioethanol more effectively by conventional yeast strains. Provided that the probability of increasing the yield of bioethanol from waste streams rich in cellulose is considered to be a promising method by researchers that aim to optimize the process stream further, especially because liquid biofuels are supposed to enlarge by 2050 to 6–8% per annum and alternative sources are necessary for achieving this goal [88]. Additionally, alternatives such as bio-oil and biomethane are investigated in macroalgal biorefinery waste streams and have opened up new pathways for investigation in research and commercialization into the energy industry.

**Figure 3.**
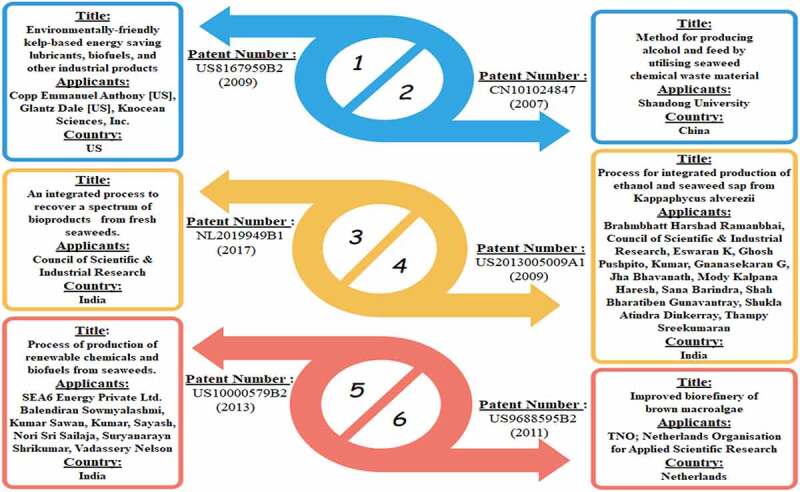
Various patent filled for biofuel generation from Macroalgae

Recently, for some years Biofuels from macroalgae have been focused on documenting few patents to commercialize the process in the industry but it was quite slow. Hence using these concepts of biotechnologies, there is no appropriate upscaling of biofuel production are available through a viable technique with proper infrastructures. Six patents describing the bioprocesses that resulted in a variety of industrially applicable biofuels and bioproducts was shown in Figure 3. Renewable and platform chemicals include biofuels such as bio-oil, bio-butanol, bio-methane, (US9688595B2) [89], fermentation sugars, sugar acids, sugar alcohols (US9688595B2), levulinic acid, hydroxymethylfurfural, and formic acid (US9452993B2) [90], and bioethanol (CN101024847, US2013005009A1) [91,92], as well as bio-fertilizer and agricultural feed (CN101024847, US2013005009A1, US10000579B2) [93]. Particularly, one such patent proposes a technique for recovering a variety of varied, industrially appealing bioproducts such as lipids, pigments, agricultural feed, and hydrocolloid agar from the red seaweed *Gracilariacorticata* (US10000579B2).

Usually, the patenting is however a laborious and expensive procedure, with the risk that the patent application will be rejected, as well as the far more difficult potential and not to patent the complete bioprocess but only a few be contingent on its individuality and creativity.It is clear in patent US8167959B2 [94], illustrating the innovation of a bioprocess-based kelp *Macrosystispyrifera* could produce bioproducts with technologies for instance non-hazardous health appliances, energy secured lubricants and its additives, biochemicals, biofuels, oil remediation sorbents and dispersants, cosmeceuticals, nutraceuticals, and pharmaceuticals products/ingredients, and aquacultural and horticultural fodder or supplements.

The research related to the field applicability of macroalgal biofuel production is increasing day by day. To fulfil the biorefinery process, furthermore investment in research and development is needed. Even though the insight of bioenergy generation from macroalgae was started in the 1970’s, the augmentation in pilot-scale and funded projects associated with biofuels generation from macroalgae was observed from 2010 onwards focused on the practical facets in the production of biofuel, food additives, and chemicals. The SeaGas Project, MacroFuels, MacroBioCrude, and GlobalSeaweed were among the initiatives that were completed [95]. However, there seems to be a lot of interest in continuing macroalgae research on a worldwide basis. Most of the government agencies perform a significant role in implementing the European such as the United States Department of Energy, the United Kingdom’s Research and Innovation, the Australian Government (Department of Industry, Science, Energy, and Resources), the New Zealand Ministry, and the European Commission. A significant number of initiatives was financed by the United States Department of Energy to develop various cultivation systems and offshore farming, however the mainstream of projects funded by the governments of the United Kingdom, Europe, Australia, and New Zealand focus on the yield of end product having the potential to commercialize a downstream by-product. Various funded projects under commercialization of the macroalgal biorefinery are shown in Table 2. Most features of the macroalgal biofuel production methods, including cultivation, harvesting, post-harvesting processing, product restoration, and implementation, and macroalgal bioproduct experiments, would be funded to educate users and aid in macroalgal biorefinery awareness. While addressing biological and engineering difficulties, it’s also important to concern about bioprocessing technologies, environmental sustainability, and constraints that might have recognized impact on policy or law [96]. Field application of various patents is a helpful indication for a better understanding of the success and development of the macroalgal biotechnology sector [21]. Patent paperwork, particularly granted applications, give important proof of the creativity, novelty, worldwide technical advancement, and economic benefit, regardless of appropriate usage of raw material in the invention.

**Table 2.** Currently ongoing funded macroalgae research projects (Source: https://arpa-e.energy.gov/technologies/projects)

S.No	Project Title	Contributors	Cost of project	Project Duration	Project inheritors	Output
**USA- UNITED STATES OF AMERICA**						
1.	Autonomous tow vessels	Advanced Research Projects Agency- EnergyU.S. Department of Energy	USD909,901	2 years 11 months 30 days(2018–2021)	C. A. Goudey and Associates	Autonomous marine tow vessel for the deployment of large-scale seaweed farming systems.
2.	Ocean energy from macroalgae	Advanced Research Projects Agency- Energy U.S. Department of Energy	USD496,483	2 years 2 months 28 days(2018–2020)	Fearless Fund	Novel system design and of large scale macroalgae ‘ranching’ using remote sensing, imaging and modeling technologies.
3.	Performance and impact of macroalgae farming	Advanced Research Projects Agency- Energy	USD995,978	2 years 5 months 30 days(2018–2020)	Macai Ocean Engineering	Tools to stimulate the biological performance of offshore macroalgal systems.
4.	Biofuels from kelp	Advanced Research Projects Agency- EnergyU.S. Department of Energy	USD2,623,787	4 years 6 months 25 days(2016–2020)	Marine Bioenergy	Development of an open ocean cultivation system for kelp biomass and bio-crude
5.	Techniques for tropical cultivation	Advanced ResearchProjects Agency- EnergyU.S. Department of Energy	USD7,515,793	4 years 11 months 29 days(2018–2023)	The MarineBiological Laboratory	Cultivation system design andbiofuels production
6.	Single point mooring array formacroalgae	Advanced ResearchProjects Agency- EnergyU.S. Department of Energy	USD4,249.547	4 years 11 months 6 days(2018–2023)	Ocean Era (formallyknown as Kampachi Farms)	seawater nutrients
7.	Continuous, high-yield kelpproduction	Advanced ResearchProjects Agency- EnergyU.S. Department of Energy	USD5,202,016	4 years 8 months 19 days(2018–2022)	Trophic/Otherlab/The University of New Hampshire	A rugged and resilient offshore sea farm with high yield and low capital cost
8.	Scalable coastal and offshoremacroalgal farming	Advanced ResearchProjects Agency- EnergyU.S. Department of Energy	USD3,132,133	4 years 10 months 20 days(2018–2023)	The University ofAlaska Fairbanks	Replicable model farms capable of cost-effective production of sugar kelp along the Alaskan coastline will be developed.
9.	Macroalgae cultivationmodeling system	Advanced ResearchProjects Agency- EnergyU.S. Department of Energy	USD1,815,529	2 years 11 months 30 days(2018–2021)	The University ofCalifornia, Irvine	Integrates an open- source regional ocean model with a fine-scale hydrodynamic model for simulating forces and nutrient flows in various seaweed farming systems.
10.	Scalable aquaculture monitoringsystem	Advanced ResearchProjects Agency- EnergyU.S. Department of Energy	USD2,003,893	2 years 11 months 29 days(2018–2021)	The University ofCalifornia, Santa Barbara	Continuously monitor all stages of seaweed biomass production, providing farm managers with farm data products to monitor farm status from outplant to harvest.
11.	Modeling tool for ocean-deployed farms	Advanced ResearchProjects Agency- EnergyU.S. Department of Energy	USD1,323,867	3 years 11 months 30 days(2018–2022)	The University ofNew England	3D computational modeling tool for macroalgae cultivation and harvest system
13.	Genome-wide seaweed studies	Advanced ResearchProjects Agency-EnergyU.S. Department ofEnergy	USD5,151,250	4 years 0 months 16 days(2018–2022)	The University ofWisconsin-Milwaukee	Development a breedingprogram for the development of macroalgae
14.	Seaweed hatchery and selective breeding technologies	Advanced ResearchProjects Agency-Energy U.S. Department ofEnergy	USD3,704,276	2 years 11 months 30 days(2018–2022)	The Woods HoleOceanographicInstitution	A selective breeding programs for sugar kelp, Saccharina latissima, and cost effectiveness of seaweed farming
15.	Monitoring macroalgae using acoustics and UUV	Advanced ResearchProjects Agency-Energy U.S. Department ofEnergy	USD2,056,621	3 years 5 months 30 days(2018–2021)	The Woods HoleOceanographicInstitution	An autonomous unmannedunderwater vehicle system for monitoring largescale seaweed farms
**UK – UNITED KINGDOM**						
16.	Teleconnected SARgassum risks across the Atlantic: building capacity for Transformational Adaption in the Caribbean and West Africa (SARTRAC)	a) UK Research and Innovation.b) Economic and Social Research Council.	£876,346(USD1,210,263)	3 years 11 months 30 days(2018–2022)	University ofSouthampton (leadresearchorganization)	Project seeks to gain anunderstanding for the reasons behind the nundation of Sargassum seaweed on the beaches of the Caribbean, Central America and West Africa.
17.	GCRF GlobalSeaweeda -Safeguarding the future of seaweed aquaculture indeveloping countries	a) UK Research and Innovation.b) Economic and Social Research Council.c) Biotechnology and Biological Sciences Research Council.d) Natural Environment Research Council.	£5,419,058(USD7,483,903)	9 years 2 months 30 days(2012–2021)	Scottish Associationfor Marine Science (lead researchorganization)	Vision of this programme is to grow the research and innovation capability of developing countries that are engaged in seaweed farming.
18.	SeaGas: Production of bio-methane from seaweed byAnaerobic Digestion (AD)	a) UK Research and Innovation.b) Biotechnology and Biological Sciences Research Council.c) Engineering and Physical Sciences Research Council.	£534,373(USD737,987)	5 years 3 months 30 days(2015–2020)	Queen’s Universityof Belfast (leadresearchorganization)	Project investigating thereplacement of grass silage in anaerobic digestion withseaweed.
19.	Oceanium: Seaweed-basedcompostable, marine safe bio- packaging	a) UK Research and Innovation.b) Innovate UK	£99,756(USD137,766)	1 years 2 months 30 days(2019–2020)	Oceanium Ltd	Bio-packaging derived fromsustainable sources seaweed to replace single use fossil-fuel based plastics
20.	Development of the automated Ooho! Machine – reducing single use plastic packaging for <100 mL liquids, condiments and cosmetics through seaweed alginate membrane	a) UK Research and Innovation.b) Innovate UK	£343,734(USD474,708)	1 years 1 months 30 days(2019–2020)	Skipping Rocks LabLimited (lead participant)Lucozade Ribena Suntory Limited, Vita Mojo InternationalLtd (participants)	automated machine to produceOohos at scale
21.	University of Exeter GCRF Global Research Translation Award: Sustainable solutionsto food security challenges.	a) UK Research and Innovation.b) Global Challenges Research Fund.c) Newton FundInnovate UK.	£621,951(USD858,159)	1 years 5 months 30 days(2019–2021)	The University ofExeter	Removal and industrialconversion of Mexico’sproblematic seaweed bloombiomass into high quality, low cost sustainable agricultural fertilizer products,
**AUSTRALIA**						
22.	Marine Bioproducts and Biotechnology Corporative Research Center (Bid inprogress)	Australian Government,Department of Industry, Science,Energy and Resources Cooperative Research Centers program	–Not Available–	–Not Available–	Flinders University	Identification and bioproduct development from marine resources such as seaweeds and microalgae.
23.	Expanding Marine BiotechProduction & Refinery Facility to meet demand	AustralianGovernment,BusinessRegional Jobs and Investment Package	AUSD600,000(USD449,586) (total grant amount)AUSD1,326,569(USD994,011) total project value)	3 years(2019–2022)	Venus Shell SystemsPty Ltd	Production and a refineryfacility to meet the increasingdemand for a range of seaweed products.
24.	Seaweed solutions for sustainable aquaculture	Australian Government,Department of Industry, Science, Energy and ResourcesCooperative ResearchCenters program	AUSD2,385,067(USD1,787,154)(Total grant amount) AUSD5,468,110(USD4,097,309)(Total project value)	3 years(2019–2022)	Tassal Group LimitedDeakin UniversityUniversity ofTasmaniaSpring Bay SeafoodsPty Ltd	Collaborative project to develop a sustainableIntegrated Multi-TrophicAquaculture (IMTA) model that supports commercial seaweed production.
25.	Seaweed production as a nutrient offset for Moreton Bay	Fisheries Research and DevelopmentCorporation	AUSD370,000(USD277,244)	1 year 2019	University of theSunshine Coast(USC)	Nutrient offset and sequestration potential of target seaweeds
**NEW ZEALAND**						
26.	Turning a pest seaweed into a high-value agricultural products	New Zealand Ministry of Primary Industry’s (MPI) Sustainable Food and Fiber Futures programme	NZD75,000(USD52,584)	1 years 6 months 30 days(2019–2020)	Waikaitu Ltd	Pest seaweed species Undaria pinnatifida into a sustainable, high-value agricultural product
27.	A cattle feed supplement to reduce greenhouse gasemissions	New Zealand Ministry of Primary Industry’s (MPI) Sustainable Food and Fiber Futures programme	NZD100,000(USD70,112)	1 year(2019–2020)	Cawthron Institute	Seaweed as a feed supplement at pilot- scale.
28.	Mussel with fucoidan as supplemented superfood – product development and clinical benefits	New Zealand Ministry of Business, Innovation and Employment High-Value Nutrition National Science Challenge	NZD803,000(USD563,003)	2 years(2020–2022)	Auckland University of Technology	A new Greenshell™ mussel Perna canaliculus as a superfood that is supplemented with fucoidan extracted from Undaria pinnatifida.
29.	Realizing the value of algae as a source of alternative protein	New Zealand Ministry of Business, Innovation and Employment Catalyst: Strategic – New Zealand – Singapore Future Foods Research Programme	NZD3,000,000(USD2,103,375)	3 years 2 months 30 days(2020–2023)	Cawthron Institute	Red seaweed Karengo and the microalga Chlorella alternative sources of Protein.
**NORWAY**						
30.	SeaBest (SME-Instrument)	EuropeanCommission (Horizon2020)	€1,660,881(USD1,963,892) (EC contribution)	1 years 11 months 27 days(2019–2021)	Seaweed EnergySolutions AS	large-scale organic seaweed-to- food cultivation.
**FRANCE**						
31.	GENetic diversity exploitation for Innovative macro-ALGalbiorefinery (GENIALG)	EuropeanCommission (Horizon2020)	€10,885,817(USD12,871,825)(EC contribution)€12,224,237(USD14,454,426)(Total budget)	3 years 11 months 30 days(2017–2020)	Center National de laRechercheScientifique CNRS	The production and sustainable exploitation of two high yielding EU species of seaweed: Saccharina latissima and Ulva spp.
**DENMARK**						
32.	Algae based climate feed additive for methane reduction in dairy cowsClimate Feed	Innovation FundDenmark	17 M DKK(USD1,908,018)	4 years(2019–2023)	DanishTechnologicalInstitute (Project manager)	Develop suitable methods for cultivating, harvesting and processing/drying seaweed into finished goods.

## 7. Challenges and limitation

**Figure 4.**
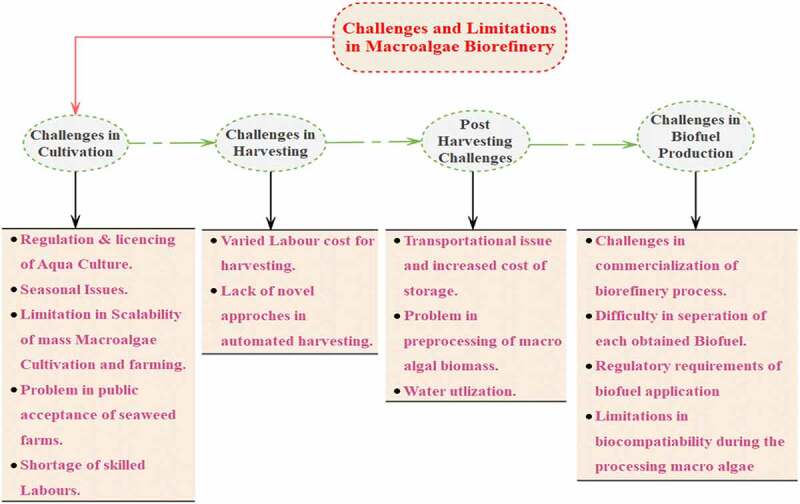
Challenges and limitations in Macroalgal biorefinery

The utmost viable and desirable fuel sources get shifted to a blue bioeconomy is the macroalgae which have emerged as promising sources for biobased products and biofuels [97]. Some of the challenges and limitations in the biofuel refinery of macroalgae are listed in Figure 4. And besides such a trove of knowledge, the advent of commercial bio-refinery technologies, which uses macroalgae mostly as feed and it might tend to be hindered with an obvious lack of practical concepts that really should be resolved before its prototype can be marketed effectively.

Growing and harvesting macroalgae is a challenge, as is the availability of an uninterrupted distribution chain for fuel sources. Other obstacles include species selectivity as well as hydrolysis, conversion, and usage of specific polysaccharides by conventional microbes. In order to provide continuous further research, the scalability of the biotechnologies used could hinder the development of macroalgal biorefineries. Many studies are presently being conducted at a lab-scale [98–100], but have uncertainty on implementation of these technologies in near future. To generate more than two products, biorefineries, which need to fully integrate each unit on a much larger scale. As a result of the scale-up, the efficiency of bioprocess and yield of bioproduct will also be reevaluated to keep track of any losses.

In the biorefinery process, the consumption of freshwater increases because it advances through its bioprocessing stages, which is also a major concern due to the global freshwater crisis [97]. In case of bioethanol production, nearly 1.5 L and 10 L of water was consumed for every liter of bioethanol produced depending on the various technique [101]. There is some other way to integrate seawater into bioprocesses. Studies have found seawater can be used in both macro-algal hydrolysis and fractionation, as well as bioethanol fermentation [99]. Some studies have shown the viability of using seawater in a particular process, and it has yet to be validated in a holistic macroalgal biorefinery process, which involves several interrelated processes and activities.

## 8. Future scope

Considering the enormous prospects for sustainable energy from this macroalgae, pretreatment enables satisfactory phase separation of the entire coastal growth must be recommended, mostly for biofuels production and for use as a food fixer, additional content, restorative, manure, and medication, boosting the biorefinery’s financial viability. Biorefinery development is based on the consistent providence and high-volume output of a suitable species of macroalgae as feedstock. But, the most significant obstacles to biorefinery advancement are the macroalgal cultivation process. Life cycle analyses and techno-economic assessments of such technologies have frequently revealed that the culture aspect of the system is perhaps the most expensive and energy-intensive, requiring further research and innovation to render macroalgal biorefineries commercially feasible. For effective development, improvements in awareness and acceptance of macroalgal development cycles, and the invention of novel efficient and suitable growing technologies (primarily for offshore cultivation) for each species of macroalgae, are critical. For each species, the energetic balance or recovery rate of the process is also necessary. It comprises reduced operational and investment expenses (such as labor, technology, and energy inputs) while enhancing and growing biomass yields and the value of potential biofuels. About 447 algae and *Spirulina spp*. production units currently exist in Europe. A variety of species, production methods and commercial applications have been identified throughout the European countries.In Europe, the harvesting of wild stocks is the predominant production system for macroalgae (68% of the production units mapped). In the case of microalgae, photobioreactors are the main production method (71%) while for *Spirulina spp*., the open ponds prevail (83%) [102]. total of 309 permits for macroalgae cultivation in Norway, of which roughly half were awarded for kelp cultivation (*S. latissima, L. digitata, A. esculenta*) with *S. latissima*at present being the commercially most important species. The total kelp production for 2017 amounted to 145 tons with a sales value of approximately 74,000 Euro [103]. International collaboration would be required to promote and develop the agricultural technology and experience of the East Asian nations (i.e., China, Korea, Japan, Indonesia, and the Philippines), which are the primary producers of macroalgal biomass for biorefinery [104] focussed to improve the macroalgal biorefinery. In 2010, these countries supplied 95% of the world’s supply. Owing to ecological circumstances such as climate, the dominant species produced in the countries differ. China and Korea cultivated 85% and 30% of the entire world production of *L. japonica* and *U. pinnatifida*, respectively [105]. *Porphyra sp*. was mostly grown in Japan, while other red algae were primarily grown in Indonesia and the Philippines [106]. East Asian countries would play a key role in expanding the amount of macroalgae produced globally for biorefinery feedstock, thanks to their decades of farming technique and experience. Various types of sensors for predicting temperature, pH etc., can be preferred in future which led to the emergence of integrated electronic technologies and the Internet for the control, monitoring, and analysis of difficulty in macroalgal growth systems. This type of technology has been implemented in microalgal cultivation system now-a-days [107]. To fractionate the ocean growing biomass, innovative and environmentally sustainable cycles are critical. According to the data gathered for this study, the most efficient and practical method for obtaining fermentable sugars is a weak corrosive pretreatment. The requirement for more eco-friendly measures brings research into pretreatments utilizing green solvents (such as water, deep eutectic solvents, and so on that when combined with effective warming frameworks, could improve the suitability of these integrated ocean growth biorefineries. There is also a demand for low-cost, earth-friendly solutions for saccharification of non-cellulosic polysaccharides. One of the most crucial obstacles is the availability of catalyst mixed drinks for particular hydrolysis of ocean growth polysaccharides. In this regard, the use of deposits from phycocolloids businesses is suggested as a feasible option, given that cellulolytic chemicals used in lignocellulosic biomass saccharification can be used. Disengagement and presentation of novel compounds from marine microbes, on the other hand, is a novel pattern that could lead to the discovery of effective proteins for saccharification of non-cellulosic kelp polysaccharides. As a result, it’s only reasonable that biotechnology improvements enable the development of microorganisms capable of hydrolyzing and aging these sugars in a combined bioprocessing setting, employing genetic modifications and metabolic designing apparatuses. The development of kelp biomass-to-biofuels measures could be aided by proper fractionated pretreatment and solidified bioprocessing.

For each macroalgae variety, which can be grown sustainably, it is necessary to determine the range of possible bioproducts and biofuels, and also the optimum, holistic, and integrated bioprocessing pathways. Such information will be essential for the bioeconomy’s long-term viability and economic benefit. Each bioprocessing step, as well as the variety of potential bioproducts, should be stored in a central database that is accessible worldwide, as this will allow the macroalgal sector to flourish.

Even if it may appear to be unduly hopeful, it is possible with strong collaboration linkages between academics and industry, as well as multidisciplinary organizations made up of cultivation experts, bioscientists, marine biologists, engineers, and social scientists. Proper Collaborations are crucial for advancement in this field. It is also critical that the bioprocessing of every macroalgal species features must be analyzed precisely for better sustainability using various innovative eco-friendly evaluation techniques once the ideal bioprocessing routes have been recognized and recorded. Some of these include life cycle assessment, energy, and energy-based models, all of which may assist progress in macroalgal biorefinery.

In offshore cultivation, proper regulation and licensing of farming in each country must be fulfilled to harvest the native macroalgal species and its affordability in order to yield better biofuels through various bioprocessing technologies. Furthermore, bio-refineries would use local species in coastal water, which have an impact on bioproducts that may vary by country. Also, the biochemical contents of macroalgae affect the potential bioproducts by creating fluctuation by taxonomic group [31]. This could have a greater impact on the bioeconomy of each country or coastal region. Furthermore, with rules varying between nations, procurement of planning authorization toward developing a biorefinery in a coastal environment may be difficult.

Due to the effects of global warming, the bioeconomy organization in a single country may alter theatrically in the coming decades. Consequently, the increased temperatures caused by climate change, studies have shown possible variations in geographical distribution and huge macroalgae in various coastline surroundings. Macroalgal distribution shifts will have an impact on macroalgal biorefinery infrastructures, their locations, jobs, and general viability in bioeconomy [108]. As a result, it is critical in the distribution of species to simulate and project the commercially important macroalgal species transition in climate change. In the decades ahead, a continued study in the development of novel macroalgal biorefineries is required mainly in the farming areas which may oppose the source of reduced feedstock or invasion of new species due to alteration in distribution [109].

In addition to the acceptability of macroalgal biorefinery by broader population and local authority, it is also important to consider the economic implications too. There might be some governmental and community groups opposition to the building of vast biorefineries at shorelines (and off-shore macroalgae growing schemes that might preferably be within close vicinity) [110]. For bio-refineries accepted by the general population, sustainable innovative strategies have been examined as well as implemented. Biotechnologies and their effects could’ve been conveyed to coastal communities prior to the biorefinery’s development [111]. Participants from academia, funders, sponsors, and the citizens could still be included in focus group meetings and/or workshops that have been accessible to all. Hence, the macroalgal sustainable biorefineries addresses the following:

- Waste biorefinery incorporated with circular bioeconomy represents a low carbon economy by involving CO_2_ sequestration which can resolve the global issues.
- Macroalgae and agriculture is additionally an expanding area of research of multi-feedstock culturing techniques focussing on the scalability of macroalgal cultivation.
- Large-scale biorefineries at coastal sites will undoubtedly provide societal benefits, including job creation, energy security, and economic development through employment
- The development of biofuels has both direct and indirect social impacts, including job creation (quality and permanence), social responsibility, and social equity, including issues such as wealth distribution to rural communities

## 9. Conclusion

In the future, a huge potential for demonstrating an integrated pattern of biofuel generation from macroalgae would be a great option. For the development of bio-fuels, bioproducts, and high-value biochemicals, research investigations have identified potential biochemical processing processes including a variety of distinct macroalgae species from all three taxonomic groupings. Its innovative potential to make a contribution to the bioeconomy and provide a sustainable renewable energy source is outlined by the intense trend in implementations of macroalgal property rights, and perhaps a rising demand of funded research projects encompasses the entire macroalgal biorefinery route. Many problems exist mostly in process of using macroalgae for producing fuels and chemicals, namely macroalgae availability and huge seasonal patterns in macroalgae biochemical and nutrient value. Certain limitations remain, such as insufficient technology and the unpredictability of the volume and quality of macroalgae biomass. The biomass of macroalgae differs by species, geographical region, and season, as well as the yields and product types generated, which are significantly reliant on processing technology. The technologies used to treat terrestrial-based biomass are generally suitable, and indeed the technique of emerging technologies or the development of new technology could well be beneficial. It’s also crucial to highlight that macroalgal biorefineries are obviously in the development stage in promoting the lab-scale techniques to an industrial scale. But, bioenergy and other macroalgal products still have the ability to impact the government’s legal and regulatory framework. These enabling states to focus on developing bioeconomic schemes and accelerate the urgency ought to prevent utilizing scarce non – renewable sources. Commercialization of such biorefineries will be possible with effective knowledge transfer and transparency between stakeholders, industry, academia, the general public, and the government. Identification of new microorganisms, technology development for genetic transformation and metabolic engineering, and process development and optimization for yield enhancement should all be prioritized to make macroalgae more effective and efficient in future. Thus, macroalgae could significantly contribute to a low-carbon economy and become the most promising biomass in future.
